# Octagonal Quasicrystal Defect Mode Laser-Based PVK: Ir(ppy)_3_ Polymer Driven by Optical Pumping

**DOI:** 10.3390/nano12091386

**Published:** 2022-04-19

**Authors:** Yuanyuan Cai, Shuai Zhang, Chenyu Wu, Zhiwei Wang, Weiran Xu, Xiao Chen, Yiquan Wang

**Affiliations:** School of Science, Minzu University of China, Beijing 100081, China; caiyuan_1988@163.com (Y.C.); s97zhang@163.com (S.Z.); wu_chenyu92@163.com (C.W.); wzw8017@163.com (Z.W.); xuweiran0820@163.com (W.X.)

**Keywords:** optical pumping, octagonal quasicrystal, low threshold

## Abstract

Based on the conjugated complex PVK: Ir(ppy)_3_, a green-emitting organic quasicrystal microcavity laser is demonstrated driven by optical pumping. The organic laser adopts a sandwich structure of DBR/organic gain membrane/output Ag-layer for the vertical oscillation and an octagonal quasi-crystal for in-plane light localization. The experimental results show that the single-mode lasing action is observed at 521 nm with an FWHM of 0.8 nm. The threshold of lasing is lowered to 0.181 μJ/cm^2^.

## 1. Introduction

Since Alan J. Heeger invented conductive polymers in 1977, research on various types of organic materials for optoelectronic devices has rapidly developed, and gradually formed an interdisciplinary application of materials, physics, chemistry, biology, and information [1,2]. In 1992, Daniel Moses realized high quantum efficiency lasing from a conducting conjugated polymer MEH-PPV in solution [3]. Solid-state polymer lasers then emerged in 1996 and have been a topic of vigorous research ever since [4,5,6]. Organic semiconductor lasers have the advantages of tunable spectra, simple fabrication, and low cost, so they hold great potential for lab-on-chip applications, bio-integration, compact display, low-cost sensing, and related areas.

Considerable effort for organic semiconductor lasers (OSLs) has been directed toward the gain media, device geometries, and fabrication techniques, etc. [7]. The results show that the main bottleneck restricting the OSLs development is large device loss, including cavity loss (light leakage, scattering and absorption) and exciton non-radiative loss (excited state absorption and exciton annihilation) that leads to a higher lasing threshold [8,9]. The compact size and high spontaneous emission coupling factor of a laser cavity make it possible to achieve a low-noise, low threshold light source.

A laser cavity as the key component applies optical feedback to establish an intense, coherent optical field inside lasers through a gain medium. Small-volume photonic cavities are generally adopted as resonators due to the capability of light localization. The optical feedback structures in OSLs contain planar microcavities, whispering gallery mode resonators, 1D distributed feedback (DFB) structures, and 2D photonic crystal resonators [10,11,12,13]. The planar microcavity is composed of a pair of metal mirrors or distributed Bragg reflectors (DBR). In 1996, Tessler first achieved lasing by using 100 nm-thick PPV film as a gain medium based on the microcavity structure, consisting of silver as the top mirror and a DBR as the bottom mirror [4]. Photonic crystals (PhCs) have the advantage of inherent flexibility in geometry, which allows fine-tuning of the defect-mode radiation pattern as well as the emission wavelength. In 1999, Painter demonstrated a 2D hexagonal photonic band-gap defect-mode laser in InGaAsP and observed the lasing with a peak emission wavelength of 1550 nm using optical pumping [14].

Compared with inorganic materials such as silicon, the refractive index of organic polymers is generally less than two. How to obtain a photonic bandgap with excellent performance under low-index contrast and realize the light localization in a 2D plane is a problem to be solved. We therefore designed an organic laser based on the quasicrystal defect microcavity. Quasicrystals, unlike periodic PhCs have been proven to offer more flexibility in modifying optical property and allow a smaller dielectric constant necessary for complete bandgaps due to the higher rotation symmetry and long-range order. For example, for TM mode, the relative permittivity threshold for eight-fold quasicrystal to produce a complete bandgap is = 1.55 (*n* = 1.24), and = 1.35 for 12-fold type [15].

In our work, an octagonal quasicrystal was adopted to achieve the lateral confinement in low-index organic light-emitting materials. A central defect microcavity was introduced to capture photons and localize inside so as to enhance the optical field. The sandwich structure of the high reflection layer/organic gain membrane/coupling output layer realized light collimation and feedback along the vertical waveguide, and finally obtained the lasing output.

## 2. Photoluminescence Spectra of PVK: Ir(ppy)_3_

**Figure 1:**
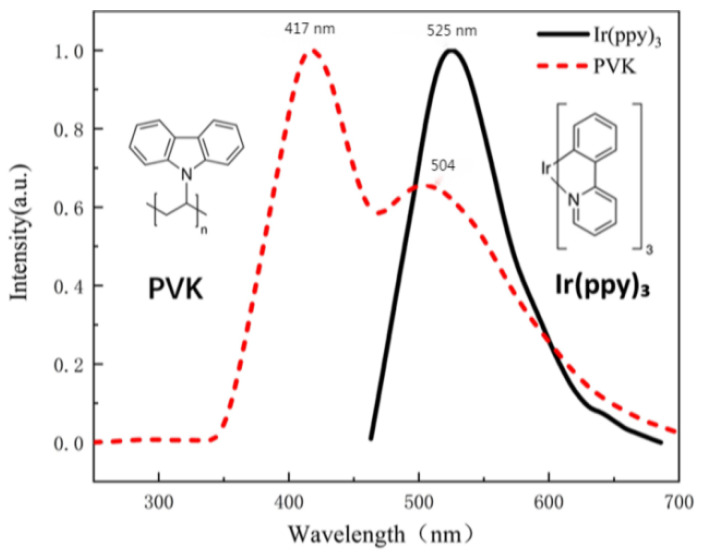
Normalized photoluminescence spectra of PVK and Ir(ppy)_3_.

Poly(9-vinylcarbazole) (PVK) is a conjugated polymer with excellent photoconductive performance. It is a popular wide-bandgap blue-emitting fluorescent host for other emissive materials such as perylene and phosphorescent dopants. PVK films have a glass transition temperature of 200 °C and a refractive index of 1.69. Tris [2-phenylpyridinato-C2,N]-iridium (III) (Ir(ppy)_3_) is a green phosphorescent emitter with high luminous efficiency [16]. Figure 1 depicts photoluminescence (PL) spectra of PVK and Ir(ppy)_3_ with the peaks at 417 nm and 525 nm, respectively.

**Figure 2:**
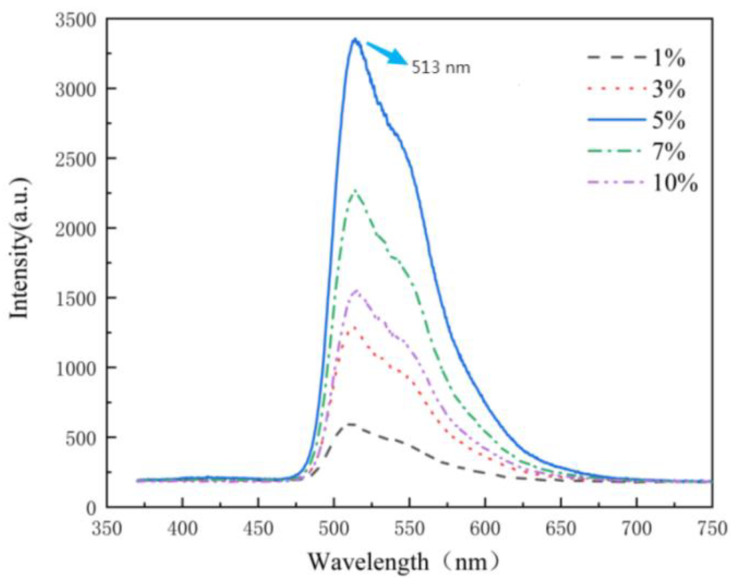
Normalized photoluminescence spectra of PVK: Ir(ppy)_3_ with different doping rates.

When Ir(ppy)_3_ is doped with the host PVK, the PL mechanism of PVK: Ir(ppy)_3_ complex is described as follows: PVK absorbs photons to generate excitons. A part of the photo-induced exciton produces a peak emission of 417 nm via the transition from the excited singlet state to the ground state, while the others transfer energy to Ir(ppy)_3_. When the doping ratio increases to 5 wt %, all singlet excitons undergo intersystem crossing into triplet excitons of Ir(ppy)_3_ due to the internal heavy atom effect, thus contributing the maximum luminescence, as shown in Figure 2. The radiation covers from 490 nm to 630 nm with the peak at 513 nm and a FWHM of 70 nm at 5 wt %. When the doping ratio of Ir(PPY)_3_ increases to more than 5%, phosphorescent materials will appear to reach luminescence saturation due to the triplet quenching of excitons, and the interaction between molecules will also lead to phosphorescent quenching. This will lead to the reduction of the luminous efficiency of the material.

## 3. Resonator

A resonant cavity defines the allowed resonant frequencies of a device (within the constraint of the organic gain medium’s emission spectrum) and the wavelength of the laser field. It also defines the spatial characteristics of the laser beam. The requirement leading to a discrete set of resonant frequencies for a given laser resonator is determined by

$$L=\frac{qc}{2nf}$$

where *f* is the resonance frequency, *q* is an integer number of half wavelength, *n* is the refractive index of a cavity, and *L* is the cavity length. For the gain medium PVK: Ir(ppy)_3_ with the emission peak *λ* = 513 nm and *n* = 1.69, the resonator achieves a single longitudinal mode output at *q* = 2, corresponding to the cavity length *L*~305 nm. Too-thin organic medium results in inadequate gain amplification, while too-thick film causes an uneven surface and insufficient ion etching in the experiment.

**Figure 3:**
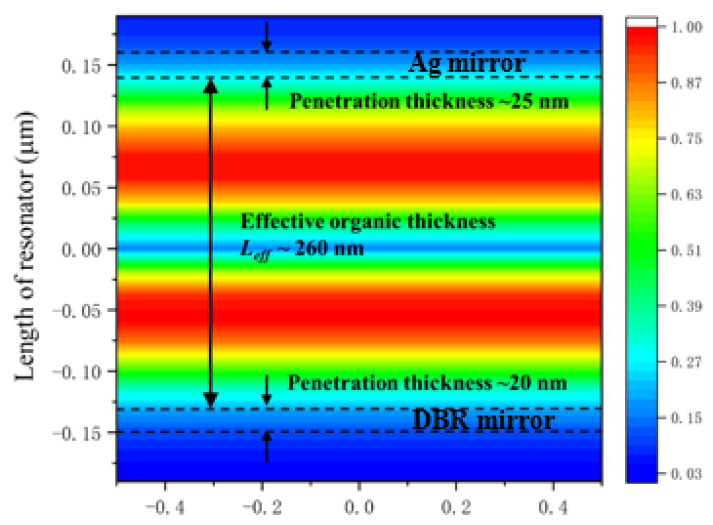
Field distribution of the wavelength 513 nm along the resonator.

A sandwich structure as a resonator consists of high reflection layer/organic gain membrane/output Ag-layer. The gain medium PVK: Ir(ppy)_3_ covers a Al_2_O_3_/TiO_2_ DBR-mirror with reflectivity up to 99.5%@500–700 nm. Next, 40 nm-thick Ag film as a partial mirror is sputtered on the top with transmissivity of 13%. It is noticed in Figure 3 that the light field partially penetrates into the top and bottom layers, and the penetration depth is about 45 nm. Therefore, the effective organic thickness *L*_eff_ is 260 nm for the device.

**Figure 4:**
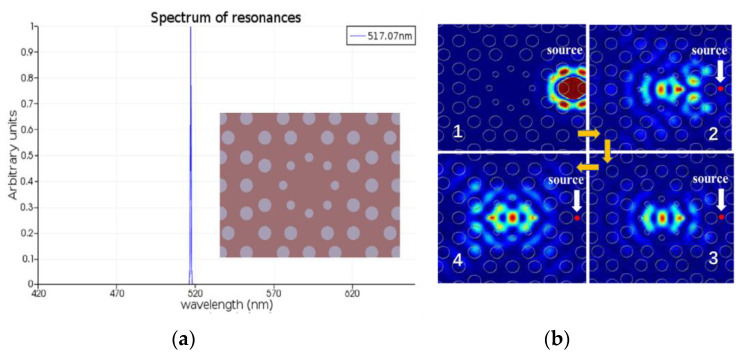
(**a**) Resonant peak output at 517 nm from an octagonal quasicrystal defect microcavity and the schematic geometry (the inset); (**b**) the evolution of intensity distribution over time with a point light source in the vicinity of a defect.

An octagonal quasicrystal defect microcavity is constructed in PVK: Ir(ppy)_3_ membrane shown in Figure 4a. The quasicrystal structure is optimized as follows: the lattice constant is 240 nm, the radius of the air rods is 70 nm, and the thickness of the organic slab is 260 nm. A point-defect cavity is introduced by two steps: removing a central air rod and then reducing the radius of the adjacent eight rods to 32 nm. The simulation result in Figure 4a demonstrates that a single defect mode occurs in the bandgap with the quality factor *Q* = 1107 (*λ* = 517 nm), close to the PL peak of PVK: Ir(ppy)_3_ complex. In addition, those neighboring photons that match the defect-supported modes around the cavity are captured and accumulated into the defect region regardless of where a light source is, which further benefits the light amplification (Figure 4b). The resonant mode is highly localized in the defect region, and photons can only escape by either tunneling through the 2D PhC or by leaking out on the slab interface at a sufficiently high angle in the vertical direction.

## 4. Experiment Results and Discussion

**Figure 5:**
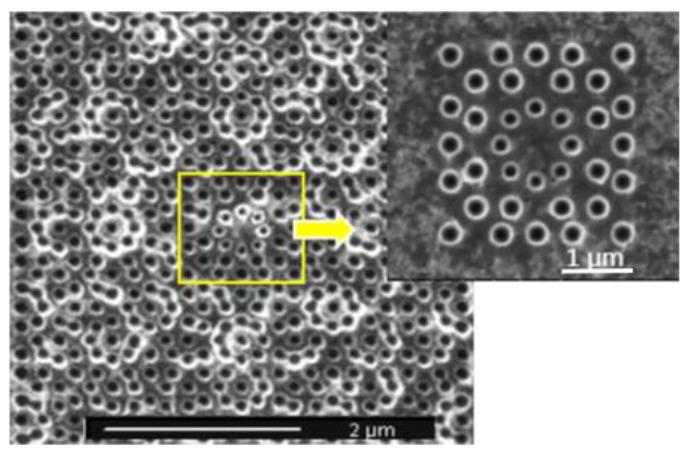
SEM image of quasicrystal structure.

In the experiment, PVK: Ir(ppy)_3_ diluted in chloroform was spin-coated onto the dielectric high-reflection film on the quartz substrate. FIB etching technology was adopted as a micro/nano-processing tool for transferring the design patterns onto the organic membrane. A scanning electron microscope (SEM) micrograph showed a top view of the fabricated microcavity (Figure 5).

**Figure 6:**
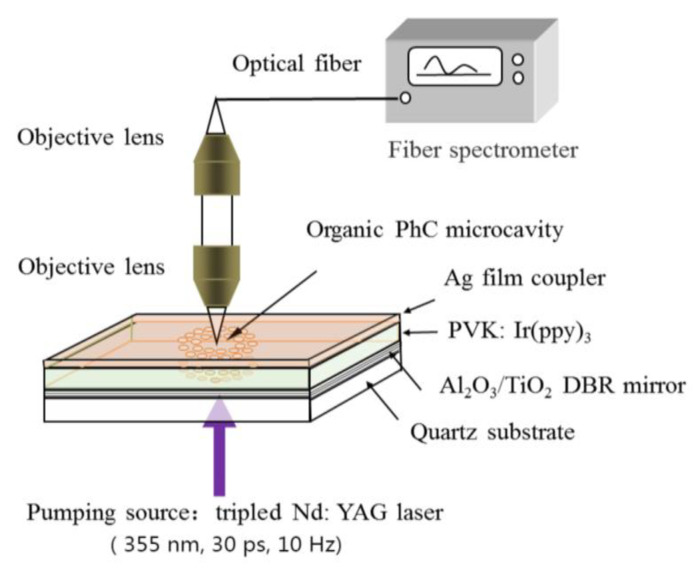
Experimental setup for the detection of an organic QPC microcavity.

**Figure 7:**
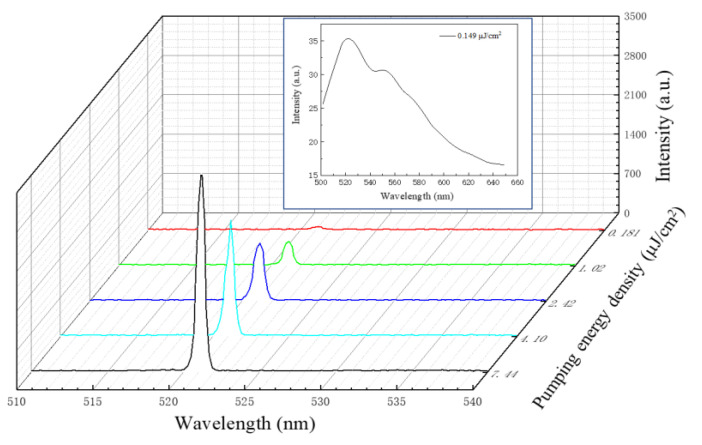
Output from octagonal quasicrystal defect-mode laser-based PVK: Ir(ppy)_3_ complex at different pumping energy density (The different color line represent different pumping energy densities).

**Figure 8:**
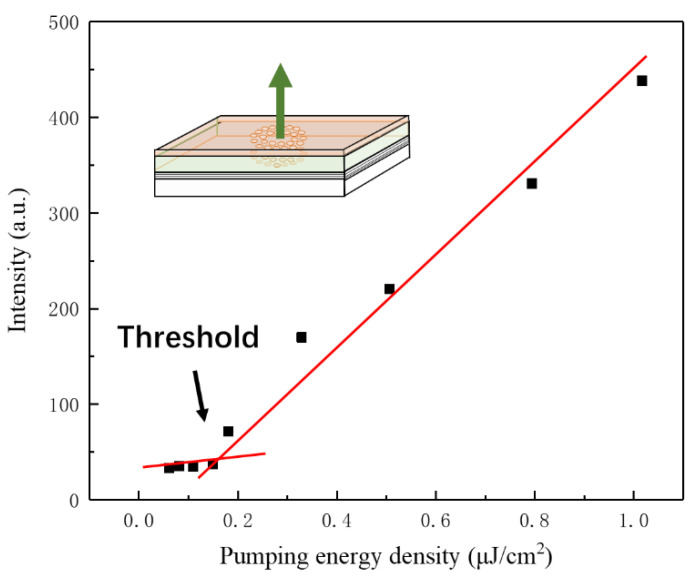
Dependence of lasing intensity on pumping energy density.

To test the optical characteristics of the prepared organic QPC microcavity laser, the device selected Nd: YAG laser pulses as the optical pumping source with 30 ps-duration at the wavelength of 355 nm, close to the absorption peak of PVK: Ir(ppy)_3_. The pumping Gaussian beam was focused to a micro-spot 250 μm in diameter on the organic membrane. Figure 6 demonstrates the schematic diagram of the experimental setup. The emission spectrum was detected by a spectrometer with the resolution of 0.4 nm. Figure 7 shows the dependence of output on the pumping-energy density. When the density was below 0.181 μJ/cm^2^, the output had a broad spectrum, which is a characteristic of spontaneous emission from PVK: Ir(ppy)_3_, as shown in the inset. Further increasing the pump energy, the photon localization and trapping by the QPC microcavity in plane and light oscillation/amplification in the vertical resonator began to modulate the emission and shape the spectrum. Once the threshold was up to 0.181 μJ/cm^2^, a dramatic change occurred in the spectrum with a sharp single-defect-mode lasing at 521 nm. The FWHM-linewidth of this mode narrowed abruptly to 0.8 nm. Later, it dominated the emission as the energy increased, thus indicating the emission was stimulated and the lasing was achieved. The output intensity was linear with the energy density after a kink at the threshold (Figure 8). The energy density threshold of Ir(ppy)_3_ was 0.181 μJ/cm^2^, which is lower than that previously reported by Chakaroun with Alq_3_:DCJBBD slab quasicrystal lasers [17], Takeuchi with an organic microcavity laser based on all the stacked polymer layers doped with pyrromethene-567 dye [18], and Wenqiang Wan with an organic laser based on DBR structure with PFO and MEH-PPV [19]. Compared with the simulated results, it is noticed the lasing peak red-shifted about 4 nm due to the etching error and inaccurate thickness.

## 5. Conclusions

In summary, based on the light-emitting characteristics of PVK:Ir(ppy)_3_ complex and the localization of quasicrystals, we designed and fabricated an optically pumped organic octagonal quasicrystal defect-mode laser with a green-emission. The laser resonator designed in the article is composed of two parts. The quasicrystal photonic crystal structure localizes and modulates photons in the horizontal direction. In the vertical direction, the selected light with specific wavelength is amplified by the different reflectivity of light at different medium interfaces, and finally the light is output in the vertical direction. The main advantages of this design are as follows: firstly, the photonic crystal structure of horizontally modulated photons is directly processed on the gain medium, namely the luminescent material, so that the intrinsic mode of the device is localized in the defect state of the photonic crystal, which can reduce the group velocity of the intrinsic mode and increase the interaction between the light field and the gain medium. Secondly, the DBR structure with high reflectivity in the green-light band is used as the substrate to improve the light reflection and oscillation in the vertical direction. The combination of the two can reduce the pumping threshold and improve the output power of the laser. The experimental results show that the single-mode lasing action using optical pumping was observed at 521.3 nm with an FWHM of 0.8 nm. The threshold of lasing was lowered to 0.181 μJ/cm^2^. The results provide ideas for the study of low-threshold lasers.

## Data Availability

Not applicable.
